# Transdiagnostic effects of combined sleep–circadian interventions on depressive symptoms and sleep outcomes in adults with mental disorders: a systematic review and meta-analysis of randomized controlled trials

**DOI:** 10.3389/fpsyt.2026.1901978

**Published:** 2026-08-06

**Authors:** Jingyu Xu, Baojuan Wang, Wenbin Zhu, Peixin Song, Haiqing Ao

**Affiliations:** 1 Guangzhou University of Chinese Medicine, Guangzhou, China; 2 First Teaching Hospital of Tianjin University of Traditional Chinese Medicine (National Clinical Research Center for Chinese Medicine), Tianjin, China; 3 The Third Affiliated Hospital of Guangzhou University of Chinese Medicine, Guangzhou, China; 4 Beijing Maikesipu Biotechnology Company Limited, Beijing, China

**Keywords:** circadian rhythm, depressive symptoms, mental disorders, sleep, systematic review and meta-analysis

## Abstract

**Objective:**

Sleep disturbance and circadian rhythm dysregulation are common in mental disorders and may contribute to depressive symptoms and functional impairment. This systematic review and meta-analysis assessed the impact of non-pharmacological interventions targeting sleep and circadian rhythms on depressive symptoms and sleep-related outcomes in adults experiencing mental disorders.

**Methods:**

A comprehensive search was conducted across several databases, including PubMed, Embase, Web of Science, the Cochrane Library, CNKI, VIP, and Wanfang, covering the period from their inception until April 12, 2026. Eligible studies were randomized controlled trials (RCTs) of adults with clinically diagnosed mental disorders receiving combined sleep–circadian interventions. The included populations covered depressive disorders, bipolar disorder, severe mental illness, attention-deficit/hyperactivity disorder, and mixed psychiatric samples. Risk of bias was assessed using the Cochrane RoB 2 tool, and evidence certainty was rated using the GRADE approach. Meta-analyses were carried out with the aid of Review Manager version 5.4. Depending on the context, standardized mean differences (SMDs), mean differences (MDs), or risk ratios (RRs) along with 95% confidence intervals (CIs) were computed.

**Results:**

Eleven RCTs were included. Sleep–circadian interventions were associated with reduced depressive symptom severity (SMD = -0.51, 95% CI: -0.72 to -0.30) and higher treatment response (RR = 1.68, 95% CI: 1.25 to 2.27). Remission also favored the intervention groups, but this estimate was based on only two trials (RR = 2.06, 95% CI: 1.09 to 3.90). Improvements were observed in insomnia severity (MD = -5.23, 95% CI: -6.41 to -4.04), sleep disturbance or sleep quality (SMD = -0.92, 95% CI: -1.19 to -0.65), sleep-related impairment (MD = -7.91, 95% CI: -9.89 to -5.93), and functional impairment (MD = -4.78, 95% CI: -6.42 to -3.15). Evidence certainty ranged from very low to moderate, and several outcomes were supported by few trials.

**Conclusion:**

Sleep–circadian interventions may improve depressive symptoms, selected sleep outcomes, and functioning in adults with mental disorders. Given the heterogeneity of diagnoses and interventions, these findings should be viewed as promising transdiagnostic evidence. Larger trials with longer follow-up are needed.

**Systematic Review Registration:**

https://www.crd.york.ac.uk/PROSPERO, identifier CRD420261412965.

## 1 Introduction

Mental disorders are a leading cause of global disability and long-term functional impairment, especially depression and bipolar disorder (DBDs) that account for most of this burden (1). Along with affective and cognitive symptoms, sleep disturbance is among the most prevalent and disabling complaints across psychiatric populations (2). Insomnia, irregular sleep–wake timing, delayed sleep phase, hypersomnia, and reduced daytime alertness are common in people with depression, bipolar disorder, schizophrenia spectrum disorders and other severe mental illness (3, 4). According to findings (5), insomnia has been found to predict the onset as well as persistence of depressive symptoms which means that insomnia can lead to mood disorders. Current insomnia guidelines also emphasize the need to identify and treat insomnia even when it occurs in the context of psychiatric comorbidity (6).

The relationship between sleep, circadian rhythm, and mental health is biologically plausible and clinically important. Circadian rhythms regulate sleep–wake timing, hormonal secretion, body temperature, light responsiveness, activity patterns, and emotional regulation (7). Disruption of these rhythms may impair mood stability, increase affective reactivity, and weaken the temporal organization of daily behavior (2, 8). In bipolar disorder, circadian instability has been linked to mood episode recurrence and dysregulated social rhythms (8). In schizophrenia and bipolar disorder, actigraphy-based evidence indicates persistent abnormalities in sleep initiation, sleep continuity, motor activity, and rest–activity rhythms even during symptomatic remission (9). These findings support the view that sleep and circadian rhythm dysregulation may represent transdiagnostic processes rather than diagnosis-specific symptoms. Several non-pharmacological interventions have been developed to target sleep and circadian dysfunction. Cognitive behavioral therapy is advised as the primary treatment option for chronic insomnia and has demonstrated advantages for patients who also experience mental health disorders (6, 10). A meta-analysis of randomized trials further indicated that improving sleep can produce meaningful improvements in depression, anxiety, stress, and overall mental health (11). Research has also examined chronotherapeutic strategies, such as wake therapy, complete sleep deprivation, bright light exposure, and advancement of sleep phase, for their possible rapid effects in alleviating depression (12–14). Trials in depression and bipolar depression have suggested that sleep deprivation or wake therapy combined with light exposure and sleep phase adjustment may accelerate symptom improvement in some patients (15–17). However, much of the existing evidence has focused on single intervention components, such as insomnia treatment alone or light therapy alone, rather than interventions designed to address sleep and circadian regulation together.

More recently, sleep and circadian dysfunction has been conceptualized as a cross-cutting treatment target across psychiatric diagnoses (18). Transdiagnostic interventions, such as the Transdiagnostic Intervention for Sleep and Circadian Dysfunction, have been designed to address multiple sleep and circadian problems within a unified framework, including insomnia, irregular sleep–wake schedules, delayed circadian timing, hypersomnia, and sleep-related daytime impairment (18, 19). Recent randomized trials have extended this approach to major depressive disorder, severe mental illness, and mixed psychiatric outpatient samples (19–21). Nevertheless, the overall efficacy of combined sleep–circadian interventions in adults with mental disorders has not been fully established. Previous reviews have generally examined broad sleep interventions, cognitive behavioral therapy for insomnia, or chronotherapy separately, leaving uncertainty about whether interventions that jointly target sleep disturbance and circadian dysregulation improve depressive symptoms, sleep outcomes, and functioning. This systematic review and meta-analysis sought to assess randomized controlled trials focused on non-pharmacological interventions for sleep and circadian rhythms in adults suffering from mental health disorders. By incorporating outcomes related to depression, sleep, and overall functioning, this research offers a more thorough evaluation of the clinical benefits associated with integrated strategies for treating sleep and circadian issues.

## 2 Methods

### 2.1 Study design and literature search

This systematic review and meta-analysis received prior registration in the International Prospective Register of Systematic Reviews (PROSPERO; CRD420261412965) and was performed following the guidelines outlined in the Preferred Reporting Items for Systematic Reviews and Meta-Analyses (PRISMA) statement (22). Two independent reviewers conducted a comprehensive literature search across multiple databases, including PubMed, Embase, Web of Science, Cochrane Library, China National Knowledge Infrastructure (CNKI), WeiPu Information Chinese Periodical Service Platform (VIP), and Wanfang database. The searches encompassed all records from each database’s inception through April 12, 2026, and there were no language restrictions applied. The search strategy combined Medical Subject Headings (MeSH) with relevant free-text terms. Detailed methodologies for the search across each database can be found in **Supplementary Table 1**.

### 2.2 Inclusion criteria

Papers were considered for inclusion if they fulfilled the specified PICOS criteria. The participant population comprised adults aged 18 years and above who were clinically diagnosed with a mental disorder. These disorders included major depressive disorder, bipolar disorder, attention-deficit/hyperactivity disorder, schizophrenia spectrum disorders, and other psychiatric conditions identified based on recognized diagnostic guidelines. Eligible interventions were non-pharmacological sleep–circadian treatments specifically designed to improve both sleep disturbance and circadian rhythm dysregulation. For this review, a combined sleep–circadian intervention was operationally defined as an intervention package that contained at least one component directly targeting sleep disturbance and at least one component intended to modify circadian timing, circadian entrainment, or daily rhythm regularity. Sleep-focused components included strategies such as sleep hygiene, stimulus control, sleep restriction or compression, sleep scheduling, and behavioral regulation of sleep continuity. Circadian-focused components included timed bright light exposure, wake therapy or total sleep deprivation, sleep phase advance, stabilization of sleep–wake timing, and reinforcement of external zeitgebers such as daytime activity, meal timing, social routines, and light–dark exposure. These interventions included transdiagnostic sleep and circadian treatment, cognitive behavioral therapy for insomnia delivered together with chronotherapeutic components, circadian reinforcement therapy, wake therapy involving total sleep deprivation followed by bright light therapy, sleep phase advance protocols, and structured programmes aimed at stabilizing daily sleep–wake schedules and circadian timing. Accordingly, the present review examined a broad family of interventions sharing combined sleep and circadian therapeutic targets, rather than a single standardized treatment model. Eligible comparators included usual care, delayed treatment, sleep hygiene education, exercise interventions, pharmacological treatment alone, or other active or inactive control conditions that did not incorporate the same combined sleep–circadian therapeutic approach. Studies were required to report at least one relevant clinical outcome related to mental health, sleep, functioning, quality of life, or adverse events. Only randomized controlled trials (RCTs) were deemed eligible.

### 2.3 Exclusion criteria

Studies were excluded if they enrolled children or adolescents only, participants without a clinically diagnosed mental disorder, or populations in which sleep disturbance or depressive symptoms were present without a psychiatric diagnosis. Non-randomized studies, observational studies, single-arm trials, case reports, case series, reviews, meta-analyses, study protocols, editorials, letters, and conference abstracts without sufficient outcome data were excluded. Trials evaluating pharmacological treatment alone, sleep hygiene alone, cognitive behavioral therapy for insomnia without a circadian component, or light therapy alone without a sleep-focused component were not included in the primary analysis. Duplicate publications from the same trial were excluded unless they reported distinct outcomes or follow-up data that were not available in the primary report. Studies were also excluded when outcome data were unavailable or could not be extracted in a form suitable for quantitative or narrative synthesis.

### 2.4 Study selection, risk of bias assessment, and certainty of evidence

The process of study identification and eligibility assessment was conducted independently by two reviewers. Following the removal of duplicate records with EndNote, titles and abstracts were screened to eliminate articles clearly deemed irrelevant. Subsequently, the full texts of studies that appeared potentially relevant were meticulously reviewed according to the predefined inclusion and exclusion criteria to ascertain final eligibility. Any differences between the two reviewers were reconciled by consensus, with a third reviewer serving as an arbitrator when deemed necessary. The methodological quality was assessed utilizing the Cochrane Risk of Bias 2 (RoB 2) tool (23). This evaluation focused on five specific domains: bias arising from the randomization process, bias resulting from deviations from the intended interventions, bias linked to missing outcome data, bias in the measurement of outcomes, and bias in the selection of reported results. Each domain, along with the overall risk of bias, was categorized as having low risk, some concerns, or high risk.

The certainty of evidence for each pooled outcome was assessed using the Grading of Recommendations Assessment, Development and Evaluation (GRADE) approach (24). Two reviewers independently rated the certainty of evidence across five domains: risk of bias, inconsistency, indirectness, imprecision, and publication bias. As all included studies were randomized controlled trials, the certainty of evidence initially started as high and was downgraded when important concerns were identified in these domains. Final certainty ratings were classified as high, moderate, low, or very low. Any disagreements between reviewers were resolved through discussion.

### 2.5 Statistical analysis

Meta-analyses were conducted utilizing Review Manager software, version 5.4. For outcomes that are continuous, the mean difference (MD) along with 95% confidence intervals (CIs) were computed when identical measurement scales were employed across studies. In cases where different instruments were used to measure clinically comparable outcomes, such as severity of depressive symptoms or overall sleep disruption, the standardized mean difference (SMD) was applied. For dichotomous outcomes like treatment response and remission, risk ratios (RRs) with 95% CIs were determined employing the Mantel-Haenszel method. Post-intervention data, or the assessment closest to the end of the intervention, were preferentially extracted for quantitative synthesis. When multiple reports were derived from the same trial, data were not entered into the same pooled analysis unless they represented distinct follow-up outcomes. The Chi-square test was employed to evaluate statistical heterogeneity, which was further quantified using the *I*² statistic. Due to anticipated clinical and methodological variations among studies—such as discrepancies in psychiatric diagnoses, intervention components, treatment environments, comparator conditions, follow-up timelines, and outcome measures—random-effects models were applied for the main pooled analyses. To address diagnostic heterogeneity, we performed an exploratory sensitivity analysis for depressive symptom severity restricted to trials primarily enrolling participants with depressive disorders. Trials including bipolar depression or mixed psychiatric samples were excluded from this analysis. A two-tailed *P* value of less than 0.05 was deemed statistically significant. To evaluate publication bias, funnel plots were intended to be utilized when there were at least ten studies available for a specific outcome; however, this analysis was not conducted as each meta-analysis included fewer than ten studies.

## 3 Results

### 3.1 Study selection and study characteristics

**Figure 1:**
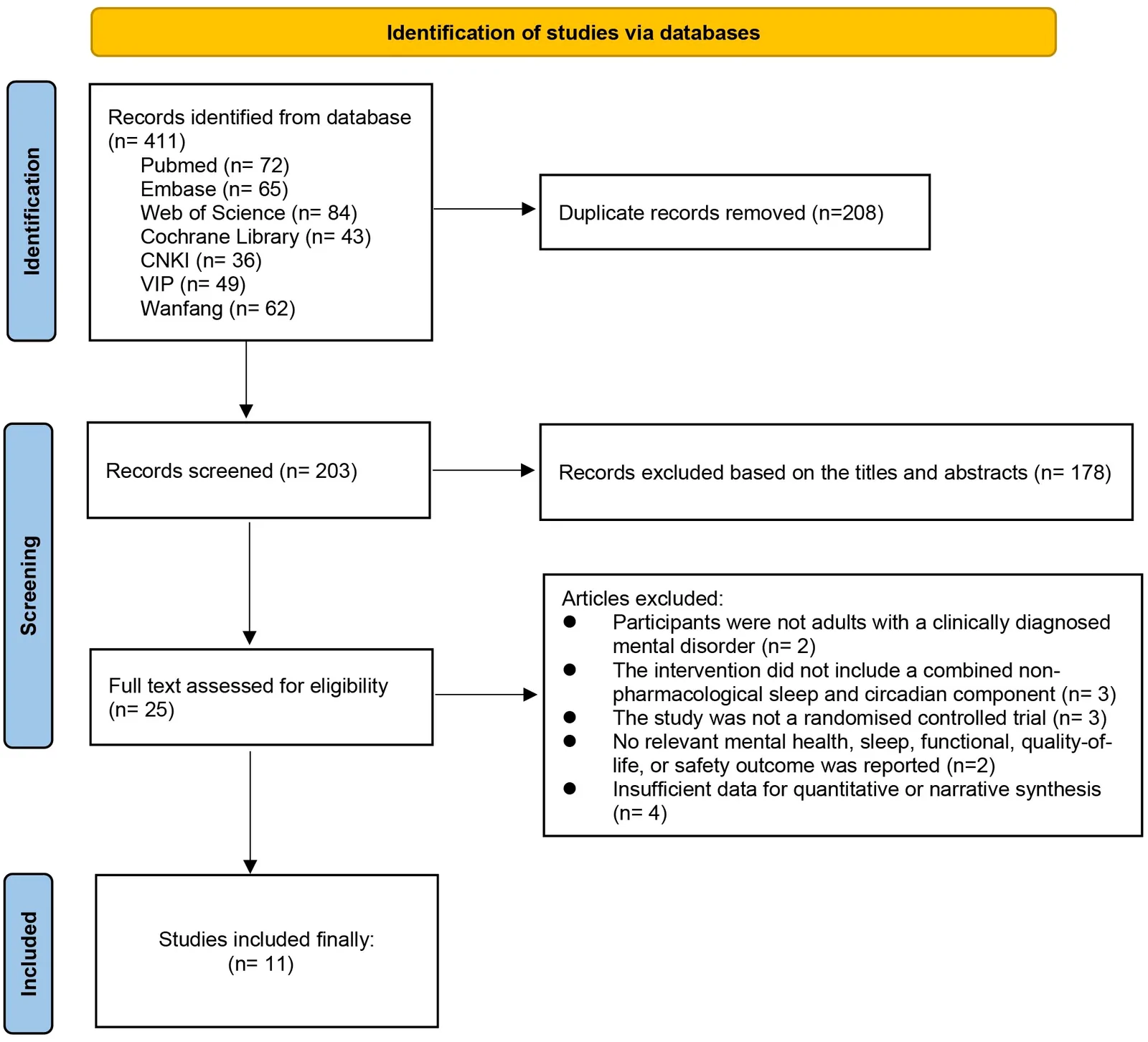
Flow diagram of study selection.

The search of the database resulted in the identification of 411 records. Following the elimination of 208 duplicates, 203 records underwent screening based on their titles and abstracts, leading to the exclusion of 178. A total of twenty-five full-text articles were evaluated to determine their eligibility. Fourteen articles were subsequently excluded because of an ineligible population, an intervention that did not include a combined non-pharmacological sleep and circadian component, a non-randomized design, absence of relevant outcome data, or insufficient data for synthesis. Ultimately, this systematic review and meta-analysis encompassed 11 distinct trials (see **Figure 1**).

**Table 1** presents a summary of the primary attributes of the studies included. The studies were published between 2009 and 2026 and were conducted in Denmark, the United States, Hong Kong, and Sweden. Most trials enrolled adults with major depressive disorder or other clinically diagnosed psychiatric conditions, including bipolar depression, severe mental illness, attention-deficit/hyperactivity disorder, and sleep or circadian rhythm disorders comorbid with psychiatric illness. Sample sizes ranged from 33 to 396 participants. The experimental interventions included wake therapy or total sleep deprivation combined with bright light therapy, sleep phase advance, sleep time stabilization, circadian reinforcement therapy, and transdiagnostic sleep and circadian interventions. As shown in **Supplementary Table 2**, the included interventions were further classified into three broad categories: chronotherapeutic interventions, circadian reinforcement or rhythm stabilization interventions, and transdiagnostic behavioral sleep–circadian programmes. Although all interventions targeted both sleep disturbance and circadian dysregulation, their core components differed substantially, indicating that the meta-analysis evaluated a broad family of combined sleep–circadian interventions rather than a single standardized treatment model. The control conditions included usual care, delayed treatment, sleep hygiene education, exercise interventions, pharmacological treatment alone, or other active or inactive control conditions. The reported outcomes included depressive symptoms, treatment response and remission, insomnia severity, sleep disturbance, sleep-related impairment, sleep diary and actigraphy parameters, functioning, quality of life, and adverse events.

**Table 1:** Characteristics of the included studies.

Author, year	Country	Study design	Study population	Sample size	Age/sex	Interventions (E/C)	Evaluation time	Outcomes
Martiny et al., 2012 (15)	Denmark	Parallel RCT	Adults with DSM-IV MDD	75 (37/38)	Age: E 46.9 (12.6); C 48.5 (11.2). M/F: E 13/24; C 18/20	E: WT + BLT + STS + duloxetine; C: exercise + duloxetine	Three main assessments: baseline, week 2, and week 9; sleep diary recorded during the 9-week treatment period.	HAM-D17, response/remission, sleep diary, WHO-5, function, AEs
Martiny et al., 2015 (25)	Denmark	RCT	Adults with DSM-IV MDD	75 (37/38)	Age: E 46.9 (12.6); C 48.5 (11.2). M/F: E 13/24; C 18/20	E: initial WT + long-term BLT/STS; C: exercise; medication changes allowed	Six assessments during follow-up: week 9, week 13, week 17, week 21, week 25, and week 29.	HAM-D17, response/remission, sleep duration/timing, WHO-5, function, AEs
Aggestrup et al., 2023 (26)	Denmark	single-blind parallel RCT	Adults with MDD after psychiatric inpatient discharge	103 (51/52); baseline 102 (50/52)	Age: E 41.8 (14.8); C 40.6 (14.8). M/F: E 19/31; C 25/27	E: CRT + MDB monitoring + IAOS; C: TAU/IAOS + MDB monitoring	Two clinical assessments: baseline and week 4; daily electronic self-monitoring throughout the 4-week intervention period.	HAM-D17, MDI, WHO-5, sleep quality/duration/timing, activity, safety
Harvey et al., 2025 (27)	USA	Hybrid effectiveness-implementation RCT	Adults with SMI and sleep/circadian dysfunction	396 (198/198)	E/C NR; TSC groups: age 38.8/42.4; M/F 31/42 and 41/83 (1 NR)	E: immediate standard or adapted TSC; C: UC-DT	Three assessments: pre-treatment, post-treatment, and 6-month follow-up.	PROMIS-SD, PROMIS-SRI, SHC, DSM-5, SDS, implementation outcomes
Yuen et al., 2021(28)	USA	Parallel RCT	Adult outpatients with nonpsychotic unipolar depression	44 (22/22)	Age: E 37.8 (14.3); C 38.8 (16.4). M/F: E 10/12; C 10/12	E: TCT (wake night + SPA + BLT); C: alternative sleep/light protocol	Baseline assessment before randomization; 12 post-randomization assessments: daily on days 1 to 7 and once weekly from week 2 to week 6.	Remission/response, SIGH-ADS/m-SIGH, QIDS, CGI, sleep log, actigraphy, AEs
Kragh et al., 2017 (16)	Denmark	Parallel RCT	Adults with ICD-10 moderate-to-severe depression	64 (32/32)	Age: E 38.4 (12.0); C 40.3 (11.5). M/F: E 16/16; C 20/12	E: WT + BLT + STS + standard treatment; C: standard treatment	Ten assessments: baseline and once weekly from week 1 through week 9; daily sleep diaries during the 9-week study period.	HAM-D17, response/remission, MDI, WHO-5, GSES, sleep diary, AEs
Wu et al., 2009(17)	USA	RCT	Adult outpatients with DSM-IV bipolar depression on medication	49 (32/17)	Age: E 39.0 (13.3); C 40.0 (14.1). M/F: E 22/10; C 7/10	E: CAT (SD + BLT + SPA) + medication; C: medication only	Repeated assessments: twice daily during the first week and once weekly from week 2 through week 7.	HRSD-19, response/remission, hypomania/mania, withdrawals
Yau et al., 2024 (20)	Hong Kong	Parallel RCT	Chinese adults with MDD and sleep/circadian dysfunction	152 (77/75)	Age: E 35.1 (11.5); C 32.8 (11.9). M/F: E 17/60; C 14/61	E: group TranS-C; C: CAU	Three assessments: baseline, week 7, and week 19.	PHQ-9, ISI, PROMIS-SD/SRI, sleep diary, HADS-A, MFI, SF-6D, SDS, AEs
Harvey et al., 2026 (29)	USA	Hybrid effectiveness-implementation RCT	Adults with SMI and sleep/circadian dysfunction	143 (78/65)	Overall age 43.5; M/F 53/90; E/C NR	E: immediate TSC delivered by Generation 2 providers; C: UC-DT	Three assessments: pre-treatment, post-treatment, and 6-month follow-up.	PROMIS-SD, PROMIS-SRI, SHC, DSM-5, SDS, implementation outcomes
Ioannou et al., 2021 (30)	Sweden	Parallel RCT	Adults admitted with a current depressive episode confirmed by MINI	33 (16/17)	Age: E 31.3 (13.1); C 29.0 (8.6). M/F: E 4/12; C 3/14	E: TSD + BLT + standard treatment; C: sleep hygiene consultation + standard treatment	Two symptom assessments: baseline and day 7; service-use follow-up at 30 and 90 days after discharge.	MADRS-S, ISI, CGI, response/remission, LOS, ED revisit/readmission, AEs
Kragh et al., 2026 (21)	Denmark	Parallel RCT	Adults with insomnia/circadian disorder comorbid with depression, ADHD/ADD, or bipolar disorder	88 (44/44)	Median age: E 27 (23-34); C 26 (24-34). M/F: E 15/29; C 18/26	E: Danish-adapted individual TranS-C; C: single-session sleep hygiene education	Three questionnaire assessments: baseline, week 2, and week 6; actigraphy and sleep diaries recorded continuously over the 6-week study period.	PSQI, ISI, WHO-5, INSPIRE, WAI, EQ-VAS, SE, SOL, WASO, NWAKE, AEs

ADD, attention-deficit disorder; ADHD, attention-deficit/hyperactivity disorder; AEs, adverse events; BLT, bright light therapy; C, control group; CAT, chronotherapeutic augmentation treatment; CAU, care as usual; CGI, Clinical Global Impression; CRT, circadian reinforcement therapy; DSM-5, Diagnostic and Statistical Manual of Mental Disorders, Fifth Edition; DSM-IV, Diagnostic and Statistical Manual of Mental Disorders, Fourth Edition; E, experimental group; ED, emergency department; EQ-VAS, EuroQol visual analogue scale; GSES, General Self-Efficacy Scale; HADS-A, Hospital Anxiety and Depression Scale-Anxiety subscale; HAM-D17, 17-item Hamilton Depression Rating Scale; HRSD-19, 19-item Hamilton Rating Scale for Depression; IAOS, intensive affective outpatient service; ICD-10, International Classification of Diseases, 10th Revision; INSPIRE, INSPIRE-O personal recovery measure; ISI, Insomnia Severity Index; LOS, length of stay; M/F, male/female; MADRS-S, Montgomery-Åsberg Depression Rating Scale-Self Assessment; MDB, Monsenso Daybuilder; MDD, major depressive disorder; MDI, Major Depression Inventory; MFI, Multidimensional Fatigue Inventory; MINI, Mini International Neuropsychiatric Interview; NR, not reported; NWAKE, number of nocturnal awakenings; PHQ-9, 9-item Patient Health Questionnaire; PROMIS-SD, Patient-Reported Outcomes Measurement Information System Sleep Disturbance; PROMIS-SRI, Patient-Reported Outcomes Measurement Information System Sleep-Related Impairment; PSQI, Pittsburgh Sleep Quality Index; QIDS, Quick Inventory of Depressive Symptomatology; RCT, randomised controlled trial; SD, sleep deprivation; SDS, Sheehan Disability Scale; SE, sleep efficiency; SF-6D, Short-Form Six-Dimension health utility index; SHC, Sleep Health Composite; SIGH-ADS, Structured Interview Guide for the Hamilton Depression Rating Scale with Atypical Depression Supplement; SMI, severe mental illness; SOL, sleep onset latency; SPA, sleep phase advance; STS, sleep time stabilisation; TAU, treatment as usual; TCT, triple chronotherapy; TranS-C, Transdiagnostic Intervention for Sleep and Circadian Dysfunction; TSC, transdiagnostic sleep and circadian intervention; TSD, total sleep deprivation; UC-DT, usual care followed by delayed treatment; USA, United States of America; WAI, Work Ability Index; WASO, wake after sleep onset; WHO-5, World Health Organization-Five Well-Being Index; WT, wake therapy.

### 3.2 Risk of bias assessment

**Figure 2:**
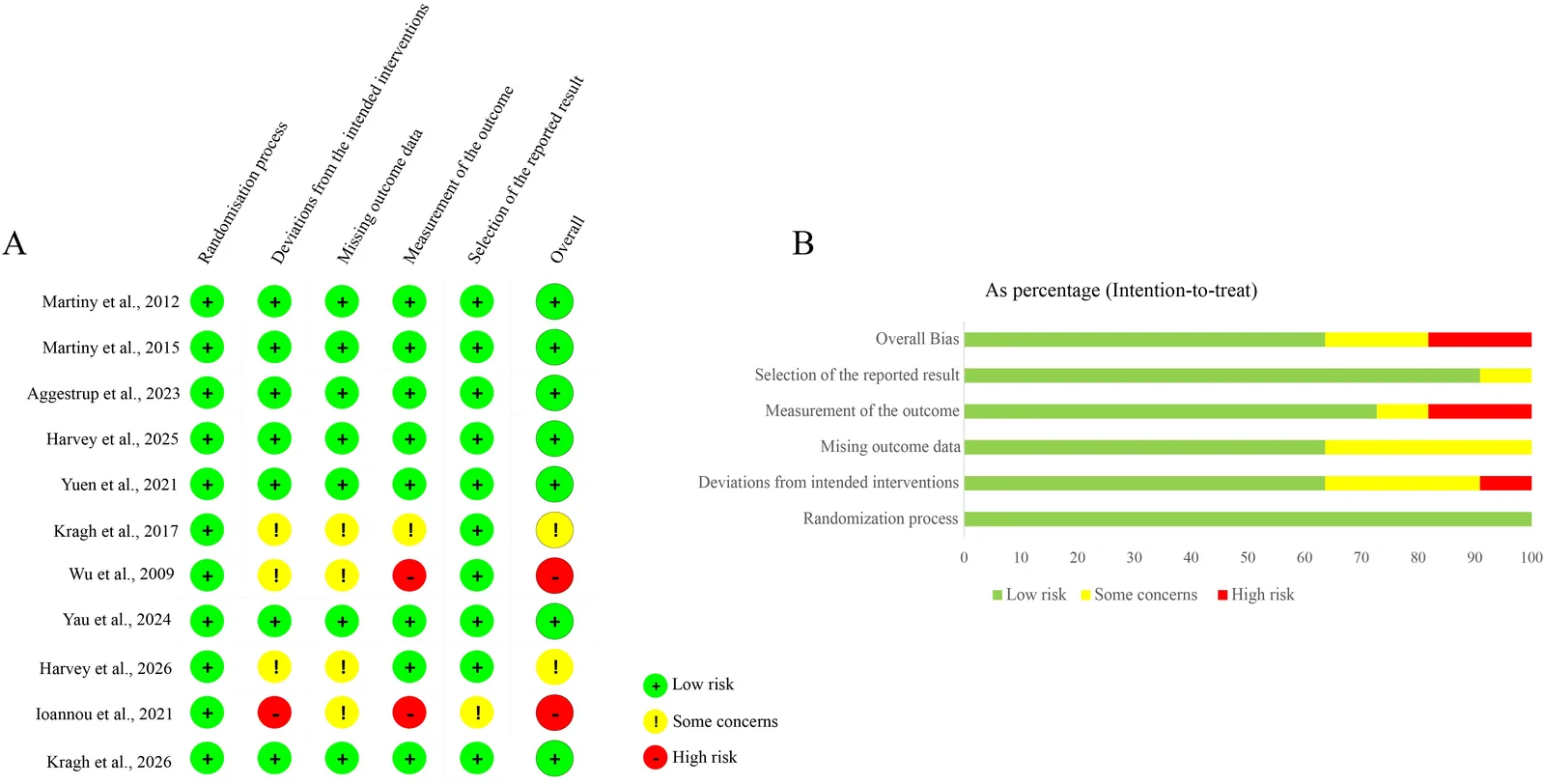
Risk-of-bias assessment of the included studies. **(A)** Risk-of-bias summary for each included study across five domains: randomization process, deviations from the intended interventions, missing outcome data, measurement of the outcome, and selection of the reported result. **(B)** Overall distribution of risk-of-bias judgements across all included studies, presented as percentages.

The evaluation of bias was conducted across five key areas: the randomization process, deviations from the planned interventions, absence of outcome data, outcome measurement, and the selection of reported results. In total, seven studies were classified as having a low risk of bias, while two studies raised certain concerns, and two were deemed to be at high risk. Every study received a low-risk rating for the randomization process. The primary areas of concern included deviations from the planned interventions, absent outcome data, and the measurement of outcomes, especially in studies where blinding was impossible or self-reported symptom assessments were utilized. The high risk of bias was primarily linked to outcome measurement and deviations from the intended intervention in chronotherapeutic studies (**Figure 2**).

### 3.3 Effects on depressive symptoms, treatment response, and remission

**Figure 3:**
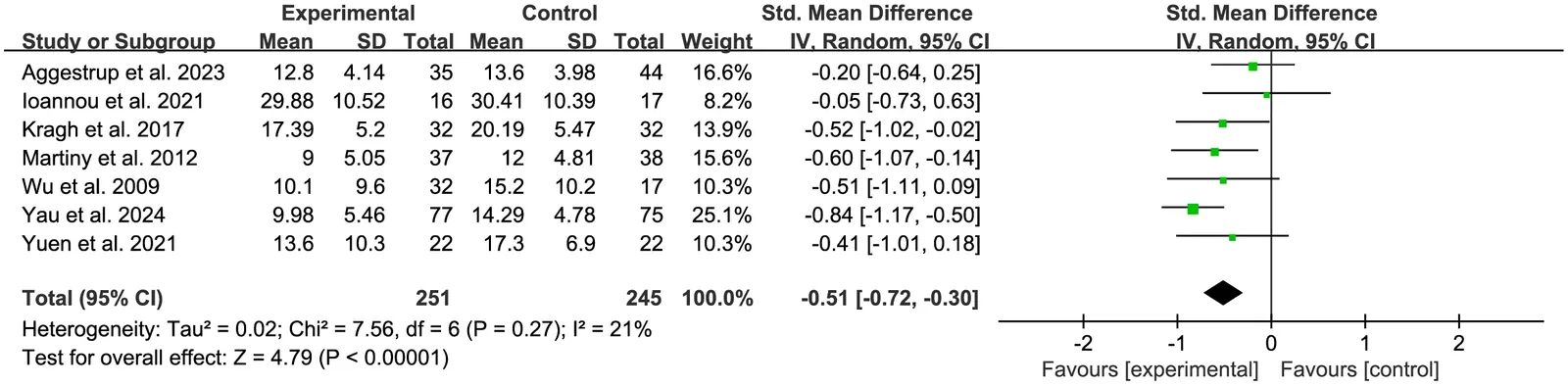
Forest plot of the effect of sleep–circadian interventions on depressive symptom severity.

Seven trials reported continuous depressive symptom scores using validated depression scales (15–17, 20, 26, 28, 30). Pooled analysis showed that non-pharmacological sleep–circadian interventions significantly reduced depressive symptom severity compared with control conditions, with a moderate overall effect size (SMD = -0.51, 95% CI: -0.72 to -0.30; *P* < 0.00001). Between-study heterogeneity was low (*I*² = 21%, GRADE = moderate certainty), suggesting that the direction and magnitude of the effect were reasonably consistent across studies despite differences in populations, intervention formats, and depression rating scales (**Figure 3**). In the exploratory sensitivity analysis restricted to depressive disorder trials, three studies were included (20, 26, 28). The pooled effect on depressive symptom severity remained statistically significant (SMD = -0.51, 95% CI -0.94 to -0.08; *P* = 0.02). Heterogeneity remained moderate to substantial (*I*² = 63%), indicating that differences in intervention format, comparator condition, and assessment timing may still have contributed to between-study variability (**Supplementary Figure 1**).

Additionally, five trials reported depressive treatment response (15–17, 28, 30). The pooled result showed that participants receiving sleep–circadian interventions were more likely to achieve a clinical response than those in the control groups (RR = 1.68, 95% CI: 1.25 to 2.27; *P* = 0.0006). No statistical heterogeneity was detected for this outcome (*I*² = 0%, GRADE = moderate certainty) (**Supplementary Figure 2A**). Two trials provided extractable remission data (15, 28). The pooled estimate favored the intervention groups, showing a higher likelihood of remission than control conditions (RR = 2.06, 95% CI: 1.09 to 3.90; *P* = 0.03), with no observed statistical heterogeneity (*I*² = 0%, GRADE = low certainty) (**Supplementary Figure 2B**). Given the small number of contributing trials, this estimate provides preliminary evidence for a possible benefit on remission rather than a definitive effect.

### 3.4 Effects on insomnia severity and sleep parameters

**Figure 4:**
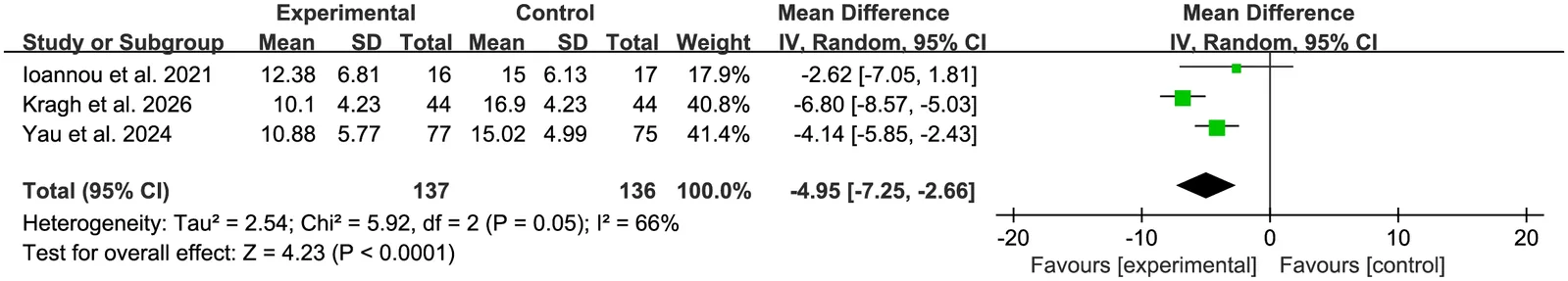
Forest plot of the effect of sleep–circadian interventions on insomnia severity.

Three studies evaluated the severity of insomnia by utilizing the Insomnia Severity Index (20, 21, 30). The combined analysis revealed a notable decrease in insomnia severity within the sleep–circadian intervention groups compared to the control groups (MD = -5.23, 95% CI: -6.41 to -4.04; *P* < 0.00001). A moderate level of heterogeneity was noted among the studies (*I*² = 66%, GRADE = low certainty), suggesting that there was variability in treatment effects across the trials (**Figure 4**). Sleep diary-derived outcomes were available for a smaller number of studies. Two trials reported sleep efficiency, and the pooled estimate did not show a clear between-group difference (MD = 5.75, 95% CI: -1.17 to 12.66; *P* = 0.10; *I*² = 63%, GRADE = low certainty) (20, 21). Sleep onset latency was reported in two trials and was shorter in the intervention groups (MD = -13.98 minutes, 95% CI: -21.61 to -6.35; *P* = 0.0003; *I*² = 0%, GRADE = low certainty) (20, 21). For wake after sleep onset, data from two trials did not indicate a significant between-group difference (MD = -6.31 minutes, 95% CI: -18.63 to 6.01; *P* = 0.32; *I*² = 41%, GRADE = very low certainty) (20, 21). Three trials reported total sleep time, and the pooled estimate favored the intervention groups (MD = 25.71 minutes, 95% CI: 12.32 to 39.11; *P* = 0.0002), with no observed heterogeneity (*I*² = 0%, GRADE = low certainty) (**Supplementary Figure 3**) (15, 20, 26). Overall, these findings suggest potential benefits for insomnia severity and selected sleep diary parameters, although the diary-based estimates were derived from few trials and were not consistent across all sleep outcomes.

### 3.5 Effects on sleep disturbance and sleep-related impairment

**Figure 5:**
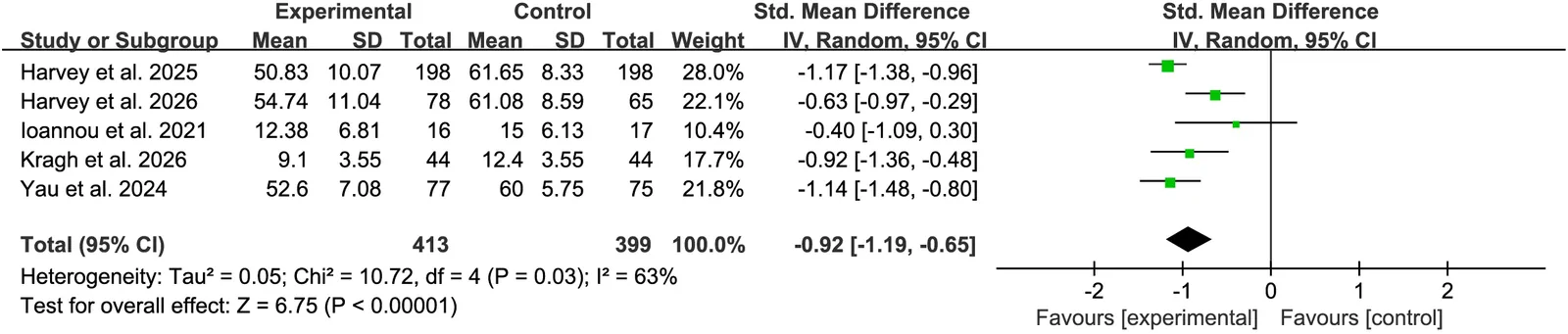
Forest plot of the effect of sleep–circadian interventions on sleep disturbance or sleep quality.

**Figure 6:**
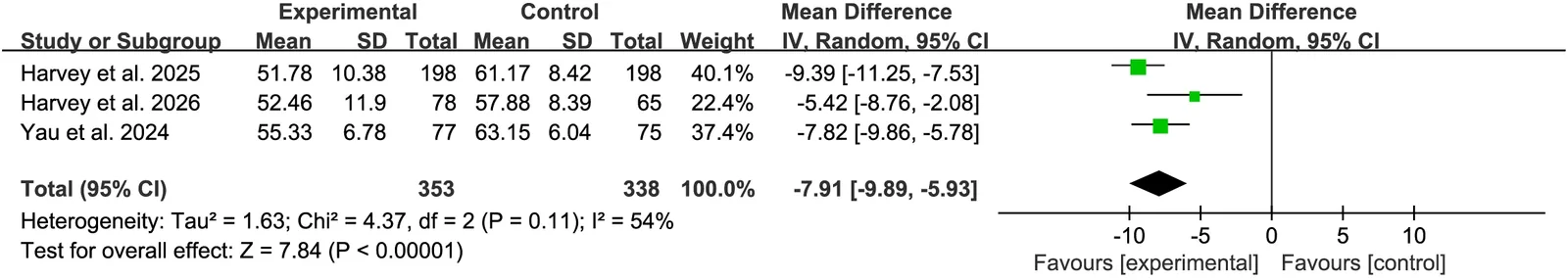
Forest plot of the effect of sleep–circadian interventions on sleep-related impairment.

Five trials reported sleep disturbance or sleep quality using validated sleep scales (20, 21, 27, 29, 30). The combined analysis revealed a notable enhancement in favor of sleep-circadian interventions when compared to control conditions (SMD = -0.92, 95% CI: -1.19 to -0.65; *P* < 0.00001). A moderate level of heterogeneity was noted among the studies (*I*² = 63%, GRADE = low certainty), which could indicate variations in sleep assessment methods, clinical populations, and formats of the interventions (**Figure 5**). Furthermore, sleep-related functional impairment was evaluated through three trials utilizing the Patient-Reported Outcomes Measurement Information System Sleep-Related Impairment (PROMIS-SRI) scale (20, 27, 29). The pooled estimate favored sleep–circadian interventions over control conditions, showing a reduction in sleep-related impairment (MD = -7.91, 95% CI: -9.89 to -5.93; *P* < 0.00001). Moderate heterogeneity was observed (*I*² = 54%, GRADE = low certainty), although the direction of effect was consistent across the included studies (**Figure 6**). However, as this result is derived from a small number of studies, it should be interpreted with caution, and further research is needed to confirm its robustness and applicability.

### 3.6 Effects on functional impairment

**Figure 7:**
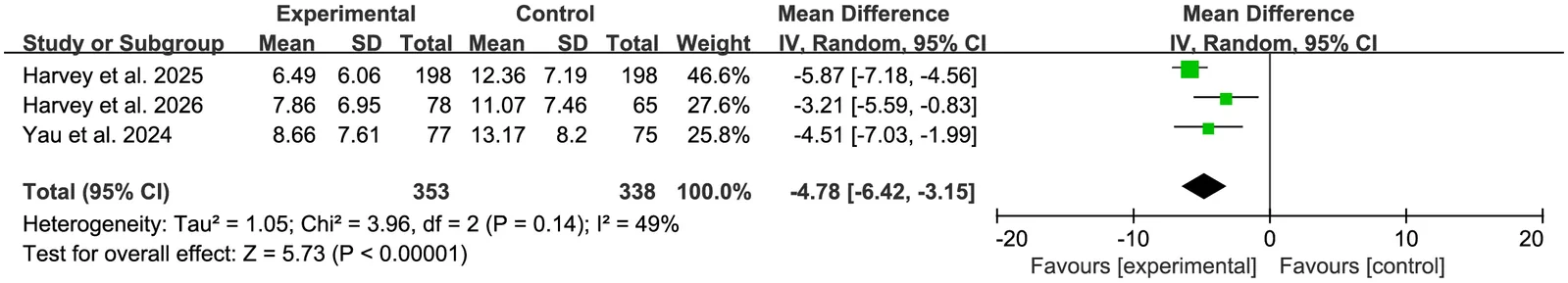
Forest plot of the effect of sleep–circadian interventions on functional impairment.

Three trials assessed functional impairment using the Sheehan Disability Scale (SDS) (20, 27, 29). The pooled estimate favored sleep–circadian interventions, showing lower functional impairment scores compared with control conditions (MD = -4.78, 95% CI: -6.42 to -3.15; *P* < 0.00001). The direction of effect was consistent across the included studies, with moderate heterogeneity observed (*I*² = 49%, GRADE = low certainty) (**Figure 7**). These findings suggest a possible benefit of sleep–circadian interventions on overall daily functioning, although the estimate was based on a small number of trials and may be influenced by differences in intervention format, clinical population, and treatment setting.

### 3.7 Follow-up duration and safety reporting

Follow-up duration and safety-related findings are summarized in **Supplementary Table 3**. The length of follow-up varied across studies, ranging from short post-treatment assessments to follow-up periods of up to 29 weeks. However, most trials primarily reported outcomes at the end of treatment or shortly thereafter, and controlled long-term evidence was limited. Safety reporting was inconsistent across studies. Reported adverse events were generally mild to moderate and included fatigue, irritability, headache, anxiety, ocular discomfort, sleep-related complaints, and difficulties with concentration or memory. Mood switching or destabilization was uncommon, but several chronotherapeutic trials included bipolar samples, and isolated cases of hypomanic or manic symptoms were reported. Overall, the available evidence was insufficient for a quantitative synthesis of adverse events or long-term durability, highlighting the need for more systematic safety monitoring and extended follow-up in future trials.

## 4 Discussion

Sleep disturbance and circadian rhythm dysregulation have been increasingly recognized as important and potentially modifiable factors across a range of psychiatric disorders (2, 8, 9). Recent studies have demonstrated associations between disruptions in the sleep–wake cycle and depressive symptoms, underscoring their relevance for prevention and early intervention (31). In addition, evidence from bipolar-spectrum populations indicates that abnormalities in sleep and circadian rhythms are already evident in individuals at high risk or in the early stages of illness (32). More broadly, research on circadian biology suggests that disruptions in these rhythms may influence emotional regulation, daytime functioning, and behavioral organization (33). In this systematic review and meta-analysis of 11 RCTs, interventions targeting both sleep and circadian processes were associated with improvements in depressive symptoms, certain sleep outcomes, sleep-related impairment, and overall functional impairment in adults with mental disorders. These findings highlight the potential clinical value of addressing sleep and circadian regulation in psychiatric treatment. However, they should be interpreted as indicative of promising transdiagnostic effects rather than conclusive evidence for disorder-specific efficacy.

The antidepressant findings of this review align with growing evidence that better sleep may confer wider benefits for mental health (10, 11). In the pooled analysis, sleep–circadian interventions led to a moderate decrease in depressive symptom severity with minimal heterogeneity. This consistency is notable because the included trials varied in diagnosis, treatment setting, intervention format, and depression scale. The mechanisms underlying this effect are likely multifactorial. Sleep consolidation may reduce affective reactivity and cognitive rumination, whereas stabilization of sleep–wake timing and circadian phase may improve daytime alertness, behavioral activation, and mood regulation (2, 6). Chronotherapeutic components, including wake therapy, sleep phase advance, and bright light therapy, may also produce rapid mood improvement through coordinated effects on sleep pressure, circadian timing, and monoaminergic systems (15, 17, 34). It is important to note that the interventions included in this review represent a diverse group rather than a single, standardized treatment approach. Some studies focused on chronotherapeutic strategies involving sleep deprivation, light therapy, and phase shifting, while others employed behavioral or transdiagnostic approaches that included elements such as sleep scheduling, stimulus control, sleep restriction or compression, sleep hygiene education, stabilization of daily routines, and regulation of light exposure. This distinction matters, as clinical guidelines and recent research suggest that individual components of sleep interventions may differ in their specific therapeutic effects (35, 36). In addition, transdiagnostic sleep–circadian interventions are typically designed to be flexible and modular, targeting a range of sleep and circadian disturbances across different clinical populations rather than focusing on a single symptom domain (19). Taken together, although the overall direction of the findings supports the effectiveness of these interventions, the current evidence does not allow for clear identification of which specific components—or combinations of components—are most responsible for the observed improvements in depressive symptoms. The increases in treatment response and remission rates further highlight the potential clinical value of these approaches, although the smaller number of studies contributing to the remission analysis warrants cautious interpretation.

The sleep-related findings further support the value of directly targeting sleep and circadian dysfunction in psychiatric populations. Participants receiving sleep–circadian interventions showed significant improvements in insomnia severity, sleep disturbance or sleep quality, and sleep-related functional impairment, suggesting that the benefits were not limited to nocturnal symptoms but also extended to perceived daytime functioning (19, 20). Analyses based on sleep diary data further indicated shorter sleep onset latency and longer total sleep time following intervention. By contrast, no clear between-group differences were observed for sleep efficiency or wake after sleep onset. Taken together, these findings suggest that sleep–circadian interventions may be more helpful for difficulties falling asleep and for alleviating the subjective burden of insomnia than for improving sleep maintenance. These results should also be interpreted in light of the clinical heterogeneity of the included samples. Sleep and circadian disturbances are relevant across diagnostic categories, but their presentation and clinical significance may differ between depressive disorders, bipolar disorder, severe mental illness, ADHD, and mixed psychiatric samples. For example, circadian sleep–wake disruption has been linked to depressive symptoms and may have implications for prevention and early intervention, while sleep and circadian abnormalities are also evident in individuals at high risk for, or in the early stages of, bipolar disorder (31, 32). In severe mental illness, sleep and circadian problems may be shaped not only by mood or psychotic symptoms, but also by medication use, social rhythm disruption, comorbidity, and reduced daytime activity (9). Therefore, the pooled sleep-related effects in this review should be viewed as transdiagnostic estimates rather than evidence of a uniform treatment response across all diagnostic groups. The moderate heterogeneity seen across several sleep outcomes may reflect variation in both the interventions themselves and the way outcomes were assessed. Some trials examined transdiagnostic behavioral programmes (21), whereas others focused on chronotherapeutic strategies such as wake therapy and bright light therapy (16, 30). In addition, sleep was assessed using different methods, including self-report questionnaires, sleep diaries, and actigraphy, and follow-up schedules also varied across studies. These methodological and clinical differences may partly explain the variation in effect sizes and limit the extent to which the results can be generalized to any single psychiatric population or sleep phenotype.

The relationship between depressive symptoms and sleep outcomes also requires cautious interpretation. These domains are closely connected in clinical practice (37, 38), and recent literature has highlighted the relevance of sleep and circadian dysfunction for depressive disorders and related psychosocial outcomes (39). However, the present meta-analysis cannot determine whether improvements in sleep mediated the reduction in depressive symptoms, whether mood improvement led to better sleep, or whether both changed through partly independent pathways. Most included trials reported study-level data and assessed depressive and sleep outcomes at similar time points, which precluded formal mediation or temporal sequencing analyses (40, 41). Moreover, some symptom measures overlap clinically, as fatigue, poor sleep, and impaired daytime functioning may contribute to both depression severity and sleep-related burden (39, 42). Thus, the findings should be interpreted as parallel improvements in related but distinct clinical domains, rather than evidence of a shared mechanism or causal pathway.

A notable finding of this review was the clear improvement in functional impairment. Sleep and circadian problems often remain even when core psychiatric symptoms have eased, and these residual disturbances can continue to interfere with daytime functioning, including work performance, social participation, and quality of life (9, 10, 43). The reduction in Sheehan Disability Scale scores suggests that the impact of sleep–circadian interventions is not limited to symptom change alone, but may also be reflected in better every day functioning. This is especially relevant for transdiagnostic approaches such as TranS-C and TSC, which are designed to address a wide spectrum of sleep and circadian problems, including insomnia, hypersomnia, irregular sleep–wake patterns, and delayed circadian phase (18–20). By targeting these overlapping disturbances, such interventions may lessen the functional burden commonly seen in people with mental disorders. From a clinical perspective, they may represent a useful adjunct to routine psychiatric care, particularly for patients whose sleep or circadian difficulties persist despite otherwise adequate treatment of the underlying disorder. Their non-pharmacological format is also an advantage, especially in individuals who are already taking antidepressants, mood stabilizers, or antipsychotic agents.

Notably, behavioral sleep interventions, such as CBT-I, are recommended as first-line treatment for chronic insomnia and may produce benefits that persist beyond the active treatment period (35, 44, 45). However, the durability of combined sleep–circadian interventions in psychiatric populations remains less certain. In the present review, most studies reported outcomes at the end of treatment or during short-term follow-up, whereas controlled long-term data were limited. This is clinically important because sleep and circadian disturbances are often chronic, recurrent, and closely linked to relapse vulnerability. Safety reporting was also inconsistent across trials. Reported adverse events were generally mild to moderate, including fatigue, irritability, headache, anxiety, ocular discomfort, and sleep-related complaints. Nevertheless, chronotherapeutic components, particularly total sleep deprivation and bright light therapy, warrant closer monitoring. Previous reviews suggest that light therapy may be useful in bipolar depression, but treatment-emergent hypomania or mood destabilization remains a relevant clinical concern, especially when bipolar samples are included (46, 47). Similarly, wake therapy and sleep deprivation have been studied as rapid antidepressant strategies, but their use requires attention to tolerability, adherence, and subsequent mood stability (48, 49). Therefore, the current evidence does not allow firm conclusions about long-term safety or sustained benefit.

Potential publication bias should also be considered when interpreting the pooled effects. Formal assessment using funnel plots or statistical tests was not appropriate because fewer than ten studies contributed to each meta-analysis. This limitation is important because small meta-analyses have low power to detect funnel plot asymmetry, and the absence of a formal test should not be taken as evidence that publication bias is absent (50). In intervention research, studies with statistically significant or favorable findings may be more likely to be published, reported in greater detail, or included in evidence syntheses than studies with null or negative results (51). This may lead to overestimation of treatment effects, particularly when the evidence base is small and when outcomes are selectively reported. For this reason, the favorable pooled estimates observed in this review should be interpreted as promising but not definitive. The GRADE assessment further supported this cautious interpretation, as the certainty of evidence ranged from very low to moderate across outcomes (52). In addition, there are other limitations to this study that should be noted. For example, the included trials differed substantially in diagnostic populations, intervention components, comparator conditions, treatment duration, and outcome measures. As a result, the pooled estimates should be interpreted as transdiagnostic effects and should not be generalized directly to any single disorder or intervention model. Second, several outcomes, including remission, sleep diary-derived parameters, sleep-related impairment, and functional impairment, were supported by a small number of studies, which limited precision and made the estimates more vulnerable to change as new evidence becomes available. Third, blinding was inherently difficult because many interventions were behavioral or chronotherapeutic, and several outcomes relied on self-report measures. Together with deviations from intended interventions, missing data, and concerns about outcome measurement, these issues may have influenced the magnitude of the pooled effects. Fourth, the available study-level data did not allow mediation analysis or temporal sequencing, so this review cannot determine whether improvements in sleep caused improvements in depressive symptoms, or whether both changed through separate but related pathways. Finally, adverse events and long-term follow-up were inconsistently reported, limiting conclusions about tolerability and durability. Future trials should use larger diagnosis-focused samples, standardized sleep, circadian, mood, and functional outcomes, objective sleep or circadian measures, longer follow-up, and prespecified adverse-event monitoring. Individual participant data analyses may also help clarify moderators of response and identify which patients are most likely to benefit.

## 5 Conclusion

This systematic review and meta-analysis suggests that combined sleep–circadian interventions may offer meaningful clinical benefits for adults with mental disorders. Improvements were observed in depressive symptoms, treatment response, certain sleep outcomes, sleep-related impairment, and overall functioning. These findings highlight the importance of addressing sleep and circadian disturbances within psychiatric care, particularly when residual sleep problems and daytime impairment persist alongside mood symptoms. However, the evidence should be interpreted as broadly supportive across conditions rather than definitive for specific disorders, given the variability in diagnoses and intervention approaches. The long-term effects and safety of these interventions, especially those involving chronotherapeutic strategies in bipolar-spectrum populations, remain uncertain. Further high-quality studies with extended follow-up, consistent outcome measures, and careful monitoring of adverse events are needed to confirm these results and determine which patients are most likely to benefit.

## Data Availability

The original contributions presented in the study are included in the article/**Supplementary Material**. Further inquiries can be directed to the corresponding author.
