# Supplementary material for: Transdiagnostic effects of combined sleep–circadian interventions on depressive symptoms and sleep outcomes in adults with mental disorders: a systematic review and meta-analysis of randomized controlled trials
Source: Front Psychiatry. 2026 Aug 6;17:1901978. doi: 10.3389/fpsyt.2026.1901978 (PMC13490127; doi:10.3389/fpsyt.2026.1901978)
Supplement: Supplementary file 1 [file SupplementaryFile1.docx]

Supplementary Material

# 1 Supplementary Figures and Tables

## 1.1 Supplementary Figures

**Supplementary Figure 1.** Sensitivity analysis restricted to depressive disorder trials for depressive symptom severity.

**Supplementary Figure 2.** Forest plots of depressive treatment response and remission. (A) Effect of sleep–circadian interventions on depressive treatment response. (B) Effect of sleep–circadian interventions on depressive remission. Risk ratios greater than 1 favour the experimental intervention. CI, confidence interval; M-H, Mantel-Haenszel.

**Supplementary Figure 3.** Forest plots of the effect of sleep–circadian interventions on sleep parameters. (A) Sleep efficiency. (B) Sleep onset latency. (C) Wake after sleep onset. (D) Total sleep time.

## 1.2 Supplementary Tables

**Supplementary Table 1.** Search strategy.

| No. | Database | Search items |
| --- | --- | --- |
| 1 | PubMed | 1. "Sleep"[MeSH Terms] OR "Sleep Wake Disorders"[MeSH Terms] OR "Insomnia"[MeSH Terms] OR "Sleep Hygiene"[MeSH Terms] OR "Sleep Deprivation"[MeSH Terms] OR "Cognitive Behavioral Therapy"[MeSH Terms]  2. sleep[Title/Abstract] OR insomnia[Title/Abstract] OR "sleep disturbance"[Title/Abstract] OR "sleep disturbances"[Title/Abstract] OR "sleep disorder*"[Title/Abstract] OR "sleep problem*"[Title/Abstract] OR "sleep quality"[Title/Abstract] OR "sleep-wake"[Title/Abstract] OR "sleep wake"[Title/Abstract] OR "sleep hygiene"[Title/Abstract] OR "stimulus control"[Title/Abstract] OR "sleep restriction"[Title/Abstract] OR "sleep compression"[Title/Abstract] OR "cognitive behavioral therapy for insomnia"[Title/Abstract] OR "cognitive behavioural therapy for insomnia"[Title/Abstract] OR CBT-I[Title/Abstract] OR "wake therapy"[Title/Abstract] OR "sleep deprivation"[Title/Abstract] OR "total sleep deprivation"[Title/Abstract]  3. #1 OR #2  4. "Circadian Rhythm"[MeSH Terms] OR "Chronotherapy"[MeSH Terms] OR "Light Therapy"[MeSH Terms] OR "Phototherapy"[MeSH Terms]  5. circadian[Title/Abstract] OR "circadian rhythm*"[Title/Abstract] OR "circadian dysregulation"[Title/Abstract] OR chronotherapy[Title/Abstract] OR chronotherapeutic[Title/Abstract] OR "light therapy"[Title/Abstract] OR "bright light"[Title/Abstract] OR "bright light therapy"[Title/Abstract] OR "blue-blocking"[Title/Abstract] OR "blue blocking"[Title/Abstract] OR "dark therapy"[Title/Abstract] OR "sleep phase advance"[Title/Abstract] OR "phase advance"[Title/Abstract] OR "social rhythm*"[Title/Abstract] OR "sleep-wake schedule"[Title/Abstract] OR "sleep time stabilization"[Title/Abstract] OR "sleep time stabilisation"[Title/Abstract] OR "circadian reinforcement"[Title/Abstract] OR "Transdiagnostic Intervention for Sleep and Circadian Dysfunction"[Title/Abstract] OR TranS-C[Title/Abstract] OR TSC[Title/Abstract]  6. #4 OR #5  7. "Mental Disorders"[MeSH Terms] OR "Depressive Disorder"[MeSH Terms] OR "Depression"[MeSH Terms] OR "Bipolar Disorder"[MeSH Terms] OR "Schizophrenia"[MeSH Terms] OR "Attention Deficit Disorder with Hyperactivity"[MeSH Terms] OR "Anxiety Disorders"[MeSH Terms]  8. "mental disorder*"[Title/Abstract] OR psychiatric[Title/Abstract] OR depression[Title/Abstract] OR depressive[Title/Abstract] OR "major depressive disorder"[Title/Abstract] OR MDD[Title/Abstract] OR bipolar[Title/Abstract] OR schizophrenia[Title/Abstract] OR psychosis[Title/Abstract] OR "severe mental illness"[Title/Abstract] OR SMI[Title/Abstract] OR anxiety[Title/Abstract] OR ADHD[Title/Abstract] OR "attention deficit"[Title/Abstract]  9. #7 OR #8  10. "Randomized Controlled Trial"[Publication Type] OR "Controlled Clinical Trial"[Publication Type] OR randomized[Title/Abstract] OR randomised[Title/Abstract] OR randomly[Title/Abstract] OR trial[Title/Abstract] OR placebo[Title/Abstract] OR "control group"[Title/Abstract]  11. #3 AND #6 AND #9 AND #10 |
| 2 | Embase | 1. 'sleep'/exp OR 'sleep disorder'/exp OR 'insomnia'/exp OR 'sleep hygiene'/exp OR 'sleep deprivation'/exp OR 'cognitive therapy'/exp  2. sleep:ti,ab,kw OR insomnia:ti,ab,kw OR 'sleep disturbance':ti,ab,kw OR 'sleep disturbances':ti,ab,kw OR 'sleep disorder*':ti,ab,kw OR 'sleep problem*':ti,ab,kw OR 'sleep quality':ti,ab,kw OR 'sleep-wake':ti,ab,kw OR 'sleep wake':ti,ab,kw OR 'sleep hygiene':ti,ab,kw OR 'stimulus control':ti,ab,kw OR 'sleep restriction':ti,ab,kw OR 'sleep compression':ti,ab,kw OR 'cognitive behavioral therapy for insomnia':ti,ab,kw OR 'cognitive behavioural therapy for insomnia':ti,ab,kw OR 'CBT-I':ti,ab,kw OR 'wake therapy':ti,ab,kw OR 'sleep deprivation':ti,ab,kw OR 'total sleep deprivation':ti,ab,kw  3. #1 OR #2  4. 'circadian rhythm'/exp OR 'chronotherapy'/exp OR 'light therapy'/exp OR 'phototherapy'/exp  5. circadian:ti,ab,kw OR 'circadian rhythm*':ti,ab,kw OR 'circadian dysregulation':ti,ab,kw OR chronotherapy:ti,ab,kw OR chronotherapeutic:ti,ab,kw OR 'light therapy':ti,ab,kw OR 'bright light':ti,ab,kw OR 'bright light therapy':ti,ab,kw OR 'blue-blocking':ti,ab,kw OR 'blue blocking':ti,ab,kw OR 'dark therapy':ti,ab,kw OR 'sleep phase advance':ti,ab,kw OR 'phase advance':ti,ab,kw OR 'social rhythm*':ti,ab,kw OR 'sleep-wake schedule':ti,ab,kw OR 'sleep time stabilization':ti,ab,kw OR 'sleep time stabilisation':ti,ab,kw OR 'circadian reinforcement':ti,ab,kw OR 'Transdiagnostic Intervention for Sleep and Circadian Dysfunction':ti,ab,kw OR 'TranS-C':ti,ab,kw OR 'TSC':ti,ab,kw  6. #4 OR #5  7. 'mental disease'/exp OR 'depression'/exp OR 'major depression'/exp OR 'bipolar disorder'/exp OR 'schizophrenia'/exp OR 'attention deficit disorder'/exp OR 'anxiety disorder'/exp  8. 'mental disorder*':ti,ab,kw OR psychiatric:ti,ab,kw OR depression:ti,ab,kw OR depressive:ti,ab,kw OR 'major depressive disorder':ti,ab,kw OR MDD:ti,ab,kw OR bipolar:ti,ab,kw OR schizophrenia:ti,ab,kw OR psychosis:ti,ab,kw OR 'severe mental illness':ti,ab,kw OR SMI:ti,ab,kw OR anxiety:ti,ab,kw OR ADHD:ti,ab,kw OR 'attention deficit':ti,ab,kw  9. #7 OR #8  10. 'randomized controlled trial'/exp OR 'controlled clinical trial'/exp OR randomized:ti,ab,kw OR randomised:ti,ab,kw OR randomly:ti,ab,kw OR trial:ti,ab,kw OR placebo:ti,ab,kw OR 'control group':ti,ab,kw  11. #3 AND #6 AND #9 AND #10 |
| 3 | Web of Science | 1. TS=(sleep OR insomnia OR "sleep disturbance" OR "sleep disturbances" OR "sleep disorder*" OR "sleep problem*" OR "sleep quality" OR "sleep-wake" OR "sleep wake" OR "sleep hygiene" OR "stimulus control" OR "sleep restriction" OR "sleep compression" OR "cognitive behavioral therapy for insomnia" OR "cognitive behavioural therapy for insomnia" OR CBT-I OR "wake therapy" OR "sleep deprivation" OR "total sleep deprivation")  2. TS=(circadian OR "circadian rhythm*" OR "circadian dysregulation" OR chronotherapy OR chronotherapeutic OR "light therapy" OR "bright light" OR "bright light therapy" OR "blue-blocking" OR "blue blocking" OR "dark therapy" OR "sleep phase advance" OR "phase advance" OR "social rhythm*" OR "sleep-wake schedule" OR "sleep time stabilization" OR "sleep time stabilisation" OR "circadian reinforcement" OR "Transdiagnostic Intervention for Sleep and Circadian Dysfunction" OR TranS-C OR TSC)  3. TS=("mental disorder*" OR psychiatric OR depression OR depressive OR "major depressive disorder" OR MDD OR bipolar OR schizophrenia OR psychosis OR "severe mental illness" OR SMI OR anxiety OR ADHD OR "attention deficit")  4. TS=(randomized OR randomised OR randomly OR trial OR placebo OR "control group")  5. #1 AND #2 AND #3 AND #4 |
| 4 | Cochrane Library | 1. MeSH descriptor: [Sleep] explode all trees  2. MeSH descriptor: [Sleep Wake Disorders] explode all trees  3. MeSH descriptor: [Insomnia] explode all trees  4. MeSH descriptor: [Sleep Deprivation] explode all trees  5. (sleep OR insomnia OR "sleep disturbance" OR "sleep disturbances" OR "sleep disorder*" OR "sleep problem*" OR "sleep quality" OR "sleep-wake" OR "sleep wake" OR "sleep hygiene" OR "stimulus control" OR "sleep restriction" OR "sleep compression" OR "cognitive behavioral therapy for insomnia" OR "cognitive behavioural therapy for insomnia" OR CBT-I OR "wake therapy" OR "sleep deprivation" OR "total sleep deprivation"):ti,ab,kw  6. #1 OR #2 OR #3 OR #4 OR #5  7. MeSH descriptor: [Circadian Rhythm] explode all trees  8. MeSH descriptor: [Chronotherapy] explode all trees  9. MeSH descriptor: [Light Therapy] explode all trees  10. MeSH descriptor: [Phototherapy] explode all trees  11. (circadian OR "circadian rhythm*" OR "circadian dysregulation" OR chronotherapy OR chronotherapeutic OR "light therapy" OR "bright light" OR "bright light therapy" OR "blue-blocking" OR "blue blocking" OR "dark therapy" OR "sleep phase advance" OR "phase advance" OR "social rhythm*" OR "sleep-wake schedule" OR "sleep time stabilization" OR "sleep time stabilisation" OR "circadian reinforcement" OR "Transdiagnostic Intervention for Sleep and Circadian Dysfunction" OR TranS-C OR TSC):ti,ab,kw  12. #7 OR #8 OR #9 OR #10 OR #11  13. MeSH descriptor: [Mental Disorders] explode all trees  14. MeSH descriptor: [Depressive Disorder] explode all trees  15. MeSH descriptor: [Bipolar Disorder] explode all trees  16. MeSH descriptor: [Schizophrenia] explode all trees  17. MeSH descriptor: [Attention Deficit Disorder with Hyperactivity] explode all trees  18. ("mental disorder*" OR psychiatric OR depression OR depressive OR "major depressive disorder" OR MDD OR bipolar OR schizophrenia OR psychosis OR "severe mental illness" OR SMI OR anxiety OR ADHD OR "attention deficit"):ti,ab,kw  19. #13 OR #14 OR #15 OR #16 OR #17 OR #18  20. (randomized OR randomised OR randomly OR trial OR placebo OR "control group"):ti,ab,kw  21. #6 AND #12 AND #19 AND #20 |
| 5 | CNKI | 1. Title/Abstract/Keyword = ("睡眠" OR "失眠" OR "睡眠障碍" OR "睡眠紊乱" OR "睡眠质量" OR "睡眠问题" OR "睡眠卫生" OR "睡眠限制" OR "刺激控制" OR "睡眠压缩" OR "认知行为治疗" OR "CBT-I" OR "睡眠剥夺" OR "觉醒治疗" OR "sleep" OR "insomnia" OR "wake therapy")  2. Title/Abstract/Keyword = ("昼夜节律" OR "生物节律" OR "日节律" OR "节律紊乱" OR "节律调节" OR "光照治疗" OR "强光治疗" OR "光疗" OR "蓝光阻断" OR "暗光治疗" OR "时间治疗" OR "时间疗法" OR "睡眠相位提前" OR "相位提前" OR "社会节律" OR "节律强化" OR "睡眠觉醒节律" OR "睡眠-觉醒节律" OR "跨诊断睡眠和昼夜节律干预" OR "TranS-C" OR "TSC" OR "circadian" OR "chronotherapy" OR "bright light therapy")  3. Title/Abstract/Keyword = ("精神障碍" OR "精神疾病" OR "精神病" OR "抑郁" OR "抑郁症" OR "抑郁障碍" OR "重性抑郁障碍" OR "双相障碍" OR "双相情感障碍" OR "焦虑障碍" OR "精神分裂症" OR "严重精神障碍" OR "注意缺陷多动障碍" OR "ADHD" OR "mental disorder" OR "depression" OR "bipolar")  4. Title/Abstract/Keyword = ("随机" OR "随机对照" OR "随机分组" OR "随机数字表" OR "对照试验" OR "临床试验" OR "RCT" OR "randomized" OR "randomised")  5. #1 AND #2 AND #3 AND #4 |
| 6 | VIP | 1. Title/Abstract/Keyword = ("睡眠" OR "失眠" OR "睡眠障碍" OR "睡眠紊乱" OR "睡眠质量" OR "睡眠问题" OR "睡眠卫生" OR "睡眠限制" OR "刺激控制" OR "睡眠压缩" OR "认知行为治疗" OR "CBT-I" OR "睡眠剥夺" OR "觉醒治疗" OR "sleep" OR "insomnia" OR "wake therapy")  2. Title/Abstract/Keyword = ("昼夜节律" OR "生物节律" OR "日节律" OR "节律紊乱" OR "节律调节" OR "光照治疗" OR "强光治疗" OR "光疗" OR "蓝光阻断" OR "暗光治疗" OR "时间治疗" OR "时间疗法" OR "睡眠相位提前" OR "相位提前" OR "社会节律" OR "节律强化" OR "睡眠觉醒节律" OR "睡眠-觉醒节律" OR "跨诊断睡眠和昼夜节律干预" OR "TranS-C" OR "TSC" OR "circadian" OR "chronotherapy" OR "bright light therapy")  3. Title/Abstract/Keyword = ("精神障碍" OR "精神疾病" OR "精神病" OR "抑郁" OR "抑郁症" OR "抑郁障碍" OR "重性抑郁障碍" OR "双相障碍" OR "双相情感障碍" OR "焦虑障碍" OR "精神分裂症" OR "严重精神障碍" OR "注意缺陷多动障碍" OR "ADHD" OR "mental disorder" OR "depression" OR "bipolar")  4. Title/Abstract/Keyword = ("随机" OR "随机对照" OR "随机分组" OR "随机数字表" OR "对照试验" OR "临床试验" OR "RCT" OR "randomized" OR "randomised")  5. #1 AND #2 AND #3 AND #4 |
| 7 | WanFang | 1. Title/Abstract/Keyword = ("睡眠" OR "失眠" OR "睡眠障碍" OR "睡眠紊乱" OR "睡眠质量" OR "睡眠问题" OR "睡眠卫生" OR "睡眠限制" OR "刺激控制" OR "睡眠压缩" OR "认知行为治疗" OR "CBT-I" OR "睡眠剥夺" OR "觉醒治疗" OR "sleep" OR "insomnia" OR "wake therapy")  2. Title/Abstract/Keyword = ("昼夜节律" OR "生物节律" OR "日节律" OR "节律紊乱" OR "节律调节" OR "光照治疗" OR "强光治疗" OR "光疗" OR "蓝光阻断" OR "暗光治疗" OR "时间治疗" OR "时间疗法" OR "睡眠相位提前" OR "相位提前" OR "社会节律" OR "节律强化" OR "睡眠觉醒节律" OR "睡眠-觉醒节律" OR "跨诊断睡眠和昼夜节律干预" OR "TranS-C" OR "TSC" OR "circadian" OR "chronotherapy" OR "bright light therapy")  3. Title/Abstract/Keyword = ("精神障碍" OR "精神疾病" OR "精神病" OR "抑郁" OR "抑郁症" OR "抑郁障碍" OR "重性抑郁障碍" OR "双相障碍" OR "双相情感障碍" OR "焦虑障碍" OR "精神分裂症" OR "严重精神障碍" OR "注意缺陷多动障碍" OR "ADHD" OR "mental disorder" OR "depression" OR "bipolar")  4. Title/Abstract/Keyword = ("随机" OR "随机对照" OR "随机分组" OR "随机数字表" OR "对照试验" OR "临床试验" OR "RCT" OR "randomized" OR "randomised")  5. #1 AND #2 AND #3 AND #4 |

**Supplementary Table 2.** Classification of combined sleep-circadian interventions included in the meta-analysis

| Study | Clinical population | Intervention category | Intervention package | Sleep-focused components | Circadian-focused components | Comparator/control | Interpretive note |
| --- | --- | --- | --- | --- | --- | --- | --- |
| Martiny et al., 2012 | Major depression/MDD treated with duloxetine; small bipolar I/II subgroup was present. | Chronotherapeutic intervention | Wake and light therapy added to duloxetine. | Three wake-therapy nights; sleep logs; sleep-time stabilization to maintain a regular sleep-wake schedule and avoid oversleeping. | Daily morning bright light therapy (10,000 lux, 30 min); mild sleep phase advance after wake-therapy nights; timing guided by morningness-eveningness score. | Daily exercise programme plus duloxetine. | Represents combined wake therapy, bright light exposure, phase advance, and sleep-wake stabilization. |
| Martiny et al., 2015 | Same major-depression trial cohort as Martiny et al., 2012; reported 20-week follow-up after the initial 9-week intervention. | Chronotherapeutic intervention (follow-up report) | Continuation/follow-up of the wake and light therapy protocol. | Continued sleep-time stabilization during the outpatient follow-up phase. | Continued daily bright light therapy during follow-up; no additional sleep-deprivation sessions during the follow-up period. | Exercise programme, with medication changes allowed in both groups during follow-up. | Follow-up report of the same RCT cohort; should not be double-counted for the same endpoint when independent samples are required. |
| Aggestrup et al., 2023 | Patients with major depression transitioning from inpatient care to outpatient affective-disorder services. | Circadian reinforcement / rhythm stabilization | Circadian reinforcement therapy (CRT) combined with electronic self-monitoring and telephone feedback. | Monitoring and stabilization of sleep timing; psychoeducation and feedback focused on sleep and mood regularity. | Strengthening zeitgeber input through regular social contact, physical activity, diet, daylight exposure, and sleep timing; intended to advance and stabilize sleep and mood rhythms. | Treatment as usual with the same electronic self-monitoring platform and weekly calls focused on data entry and safety monitoring. | Primarily targets rhythm regularity and zeitgeber reinforcement rather than a manualized CBT-I programme. |
| Harvey et al., 2025 | Adults with serious mental illness and sleep/circadian disturbance in community mental health settings; diagnostically mixed sample. | Transdiagnostic behavioural sleep-circadian programme | Adapted or standard Transdiagnostic Intervention for Sleep and Circadian Dysfunction (TSC) delivered by community mental health providers. | Sleep education and individualized behavioural modules addressing sleep disturbance, sleep-related impairment, time in bed, sleep-wake routines, and sleep-related worry. | Modules supporting regular sleep-wake timing, daily routines, light-dark exposure, activity scheduling, and circadian rhythm stabilization. | Usual care followed by delayed TSC. | Effectiveness-implementation trial; evaluates adapted versus standard delivery formats within a broad SMI population. |
| Yuen et al., 2021 | Adults with unipolar depression, including MDD, persistent depressive disorder, or unspecified depressive disorder. | Chronotherapeutic intervention | Triple chronotherapy protocol. | One wake night/total sleep deprivation; prescribed sleep-wake schedule; sleep hygiene education. | Sleep phase advance combined with morning bright light exposure (10,000 lux, 30 min). | Alternative protocol without wake night; sleep schedule based on chronotype and light-box sitting with amber goggles. | Acute efficacy trial; the comparator was not fully inert because it included structured sleep scheduling and light-box procedures. |
| Kragh et al., 2017 | Adults hospitalized with moderate-to-severe depression; small bipolar depression subgroup included. | Chronotherapeutic intervention | Wake and light therapy added to standard inpatient treatment. | Three wake-therapy nights during the first week; sleep diaries; sleep-time stabilization and sleep-hygiene guidance to regularize the sleep-wake cycle. | Daily bright light therapy (10,000 lux, 30 min) for 9 weeks; scheduled timing based on chronotype; sleep phase advance after wake-therapy nights. | Standard treatment, including individualized pharmacotherapy and usual inpatient care. | Chronotherapeutic adjunct in a more severe inpatient depression sample. |
| Wu et al., 2009 | Adults with bipolar disorder experiencing a current major depressive episode. | Chronotherapeutic intervention | Chronotherapeutic augmentation added to pharmacotherapy. | Total sleep deprivation/sleep deprivation procedure with subsequent scheduled recovery sleep. | Bright light therapy and sleep phase advance delivered with mood stabilizer/antidepressant treatment. | Medication treatment alone/usual pharmacological management. | Bipolar-depression-specific chronotherapy trial; relevant for safety and mood-switching considerations. |
| Yau et al., 2024 | Adults with MDD and at least one sleep or circadian problem. | Transdiagnostic behavioural sleep-circadian programme | Group-based TranS-C delivered over 6 weeks. | Sleep/circadian education, regular sleep-wake scheduling, wind-down and wake routines, sleep efficiency strategies, cognitive and behavioural work on sleep beliefs and sleep-related impairment. | Light management, regularization of daily rhythms, chronotherapy-informed phase adjustment, activity scheduling, and rhythm-stabilizing routines. | Care as usual. | Manualized group behavioural programme integrating CBT-I, interpersonal/social rhythm principles, chronotherapy, and motivational enhancement. |
| Harvey et al., 2026 | Adults with serious mental illness and sleep/circadian disturbance treated in community mental health settings; diagnostically mixed sample. | Transdiagnostic behavioural sleep-circadian programme | Adapted or standard TSC implemented through a train-the-trainer model. | Behavioural modules targeting sleep disturbance, sleep-related impairment, sleep routines, time in bed, and sleep-related anxiety. | Regularization of sleep-wake timing and daily routines; attention to light-dark exposure, activity patterns, and circadian stabilization. | Usual care followed by delayed TSC. | Implementation-focused trial testing whether TSC effects could be retained when training was delivered through local trainers. |
| Ioannou et al., 2021 | Adults hospitalized with a current depressive episode, including MDD, mixed anxiety-depression, and bipolar depression. | Chronotherapeutic intervention | Total sleep deprivation followed by bright light therapy. | One night of total sleep deprivation; sleep-related guidance embedded in the protocol. | Six mornings of bright light therapy (10,000 lux, 30 min) timed according to morningness-eveningness score. | Short sleep-hygiene consultation plus standard inpatient psychiatric treatment. | Pragmatic inpatient trial with a short primary follow-up; did not include a full sleep phase advance or extended sleep-time stabilization protocol. |
| Kragh et al., 2026 | Outpatients with sleep problems and depression, attention-deficit disorder/ADHD, or bipolar disorder. | Transdiagnostic behavioural sleep-circadian programme | Individual Danish adaptation of TranS-C delivered over six sessions. | Sleep diary, sleep restriction/compression, stimulus control, sleep hygiene, cognitive techniques, relaxation, and relapse prevention. | Phase advance strategies, morning light exposure when indicated, regularization of social rhythms and sleep-wake timing. | Sleep-hygiene education/waitlist before receiving TranS-C. | Transdiagnostic outpatient sleep-circadian intervention; primary outcomes focused on insomnia severity and sleep quality. |

**Supplementary Table 3.** Follow-up duration, adverse events, and reported mood switching or destabilization across included studies.

| Study | Clinical population | Follow-up duration | Adverse events / tolerability reporting | Mood switching or destabilization |
| --- | --- | --- | --- | --- |
| Martiny et al., 2012 | Mainly major depressive disorder; small bipolar I/II subgroup | 9 weeks | Wake therapy plus BLT and sleep time stabilization was generally feasible. Reported events included anxiety/panic attacks requiring benzodiazepines in the wake group, one hypoglycemic event, tearing during light therapy, and Achilles tendon pain in the exercise group. | No mania reported. One hypomanic episode occurred in each group. |
| Martiny et al., 2015 | Mainly major depressive disorder; small bipolar I/II subgroup | 29 weeks total; 20-week follow-up phase | UKU-assessed adverse symptoms were reported during follow-up; concentration and memory problems, increased sweating, and reduced sexual desire were common, with no clear group difference. Dropout was somewhat higher in the chronotherapeutic group. | One manic episode leading to dropout and additional hypomanic events were reported in the exercise group during follow-up. |
| Aggestrup et al., 2023 | Major depressive episode after discharge; bipolar disorder and psychotic depression excluded | 4 weeks | Eight rehospitalizations occurred (CRT 3; TAU 5). Two nonfatal overdose events related to suicidal ideation occurred in TAU. Some CRT participants reported that the program was too intense or burdensome. | No mania or hypomania reported; bipolar disorder was excluded. |
| Harvey et al., 2025 | Serious mental illness with sleep/circadian disturbance; mixed diagnoses | Post-treatment at 4 or 8 weeks; 6-month follow-up collected for immediate TSC | Suicidal ideation/behavior and related safety outcomes were assessed, but adverse events were not systematically pre-specified or comprehensively reported. Treatment dropout was higher with the standard than adapted version. | Mood switching or destabilization not specifically reported. |
| Yuen et al., 2021 | Unipolar depression | Primary acute outcome at 1 week; trial assessments up to 6 weeks | Adverse-event burden during week 1 was similar between groups. Reported events included nausea, headache, eye discomfort/visual problems, and other mild symptoms; no agitation/excitement was observed in the triple chronotherapy group. | No mania or hypomania reported. |
| Kragh et al., 2017 | Moderate-to-severe depression; small bipolar subgroup | 9 weeks | Wake therapy was associated with fatigue, irritability/restlessness, sensitivity, concentration and memory difficulties, and headache. One participant withdrew because of increased anxiety; one self-harm event occurred during week 2. | No mania or hypomania developed during the intervention. One participant was excluded before intervention because of hypomanic symptoms. |
| Wu et al., 2009 | Bipolar depression | 7 weeks | Five CAT participants discontinued early for reasons including relocation, medication intolerance, and difficulty adhering to the follow-up protocol. No adverse events were reported in the medication-only group. | Two CAT participants had transient hypomanic switches, both resolving within 24 hours without additional medication. |
| Yau et al., 2024 | Major depressive disorder with sleep/circadian problems | Post-treatment at 7 weeks; 12-week follow-up at week 19 | No adverse reactions were reported during the study. Treatment dropout occurred in both groups. | No switching or mood destabilization reported. |
| Harvey et al., 2026 | Serious mental illness with sleep/circadian disturbance; mixed diagnoses | Post-treatment at 4 or 8 weeks; 6-month follow-up collected for immediate TSC | Suicidal ideation, suicidal behavior, and substance-use outcomes were explored; systematic adverse-event reporting was not clearly described. Treatment dropout during treatment was higher with standard TSC than adapted TSC. | Mood switching or destabilization not specifically reported. |
| Ioannou et al., 2021 | Inpatients with current depressive episode; included unipolar, mixed anxiety-depression, and bipolar depression | 1 week; planned 10-week follow-up discontinued | Half of TSD/BLT participants reported no adverse reactions. Reported events included fatigue, worsened insomnia, eye fatigue, restlessness, and tonsillitis. One participant discontinued because of anxiety and increased self-harm thoughts. | No manic switch or serious adverse event reported. |
| Kragh et al., 2026 | Outpatients with sleep problems and depression, ADHD/ADD, or bipolar disorder | 6 weeks | Adverse events were assessed only in the intervention group. Reported events included fatigue, headache, inner turmoil, and eye irritation related to light therapy. | Mood switching or destabilization not reported. |

ADHD/ADD = attention-deficit/hyperactivity disorder/attention-deficit disorder; AE = adverse event; BLT = bright light therapy; CAT = combined chronotherapeutic augmentation therapy; CRT = circadian reinforcement therapy; NR = not reported; TAU = treatment as usual; TSC = Transdiagnostic Intervention for Sleep and Circadian Dysfunction; TSD = total sleep deprivation; UKU = Udvalg for Kliniske Undersogelser side-effect rating scale. Safety and follow-up reporting differed across trials; absence of reporting should not be interpreted as absence of events.
